# Extracellular vesicles in COVID-19 prognosis, treatment, and vaccination: an update

**DOI:** 10.1007/s00253-023-12468-6

**Published:** 2023-03-14

**Authors:** Adrián Hernández-Díazcouder, César Díaz-Godínez, Julio César Carrero

**Affiliations:** 1 Departamento de Inmunología, Instituto Nacional de Cardiología Ignacio Chávez, Ciudad de México, México; 2 Departamento de Ciencias de La Salud, Universidad Tecnológica de México (UNITEC), Estado de México, Los Reyes, México; 3 Departamento de Inmunología, Instituto de Investigaciones Biomédicas, Universidad Nacional Autónoma de México (UNAM), 04510 Ciudad de México, México

**Keywords:** COVID-19, SARS-CoV-2, Extracellular vesicles, Treatment, Prognosis, Vaccination

## Abstract

**Abstract:**

The lethality of the COVID 19 pandemic became the trigger for one of the most meteoric races on record in the search for strategies of disease control. Those include development of rapid and sensitive diagnostic methods, therapies to treat severe cases, and development of anti-SARS-CoV-2 vaccines, the latter responsible for the current relative control of the disease. However, the commercially available vaccines are still far from conferring protection against acquiring the infection, so the development of more efficient vaccines that can cut the transmission of the variants of concerns that currently predominate and those that will emerge is a prevailing need. On the other hand, considering that COVID 19 is here to stay, the development of new diagnosis and treatment strategies is also desirable. In this sense, there has recently been a great interest in taking advantage of the benefits offered by extracellular vesicles (EVs), membrane structures of nanoscale size that carry information between cells participating in this manner in many physiological homeostatic and pathological processes. The interest has been focused on the fact that EVs are relatively easy to obtain and manipulate, allowing the design of natural nanocarriers that deliver molecules of interest, as well as the information about the pathogens, which can be exploited for the aforementioned purposes. Studies have shown that infection with SARS-CoV-2 induces the release of EVs from different sources, including platelets, and that their increase in blood, as well as some of their markers, could be used as a prognosis of disease severity. Likewise, EVs from different sources are being used as the ideal carriers for delivering active molecules and drugs to treat the disease, as well as vaccine antigens. In this review, we describe the progress that has been made in these three years of pandemic regarding the use of EVs for diagnosis, treatment, and vaccination against SARS-CoV-2 infection.

**Key points:**

*• Covid-19 still requires more effective and specific treatments and vaccines.*

*• The use of extracellular vesicles is emerging as an option with multiple advantages.*

*• Association of EVs with COVID 19 and engineered EVs for its control are presented.*

## Introduction

In recent years, viral diseases became serious public health problems. Ebola hemorrhagic fever (Onyango et al. 2004), influenza AH1N1 (Girard, et al. 2010), severe acute respiratory syndrome (SARS, Demmler and Ligon 2003), or Middle East respiratory syndrome (MERS, Fehr et al 2017) are examples of viral diseases triggering epidemics and pandemics that have alerted health institutions worldwide in the last two decades. The rapid spread of viral diseases, especially respiratory ones, must be monitored and reported before the cases increase to uncontrollable numbers, which is promoted by factors such as population mobility and globalization (Wu et al. 2017). A new respiratory human virus responsible for the current pandemic emerged in late 2019 in Wuhan, China (Zhu et al. 2020). Subsequent analyses identified it as a member of the coronavirus family and it was formally assigned the name of SARS-CoV-2 (severe acute respiratory syndrome coronavirus 2; Coronaviridae Study Group of the International Committee on Taxonomy of Viruses 2020) and the associated disease, as COVID-19 (coronavirus disease 2019, World Health Organization 2020). COVID-19 is characterized by such symptoms as fever, cough, difficulty breathing, fatigue, body aches, headache, loss of smell or taste, diarrhea, among others (Center for Disease Control and Prevention 2022). The severity of the disease depends on several factors: age (> 45 years old), gender (male), presence of chronic diseases (diabetes, hypertension, obesity), and vaccination (Rahman and Sathi 2021; Wingert et al. 2021), which makes non-vaccinated elder people the priority group to protect (Zhang et al. 2022A, B). In less than 3 years, COVID 19 became a serious public health problem that took the lives of almost 7 million people worldwide (https://coronavirus.jhu.edu/map.html).

Since the early stages of COVID-19 pandemic, treatment strategies have been sought to reduce its mortality and the risk of severe infection. Currently, the treatment consists of palliative therapy using non-steroid anti-inflammatory and analgesic drugs (Moore et al. 2021; Leal et al. 2021), antiviral drugs, e.g. paxlovid, remdesivir, or molnupiravir (Center for Disease Control and Prevention 2022-B), and monoclonal antibodies (Hwang et al. 2022). Other less conventional treatments have also been used in the quest for better medical care, including hyperimmune serum (Zylberman et al. 2020) and drugs utilized in other pathologies such as cancer (El Bairi et al. 2020). Different parameters are currently used to determine the prognosis of patients, mainly those who are hospitalized (Ruscica et al. 2021; Zemlin et al. 2022), but the acquisition of more data in correlation to the severity of the disease are pivotal to determine the course of treatment and to perform early intervention.

The implementation of mass vaccination campaigns against COVID-19 worldwide has successfully resulted in a decrease in the numbers of cases and hospitalizations reported in different countries (Wang et al. 2022B). Nevertheless, the high mutation capacity of the virus has given rise to the variants that continue to generate waves of contagion, mainly related to the evasion of the immune response generated after vaccination or infection (Harvey et al. 2021). This once again puts health systems on alert in the search for treatment and vaccination alternatives that will make it possible to stop future outbreaks.

“Extracellular vesicles” (EVs) is the term used to define a family of membrane bodies released from almost all cell types having an important role in cellular communication (van Niel et al. 2022; Díaz-Godínez et al. 2022). According to their origin, size, and cargo, Evs are classified into three categories: exosomes, microvesicles, and apoptotic bodies (Doyle and Wang 2019). Exosomes (30–100 nm in size) originate from the multivesicular bodies and are released after fusion with the plasma membrane containing constant markers such as tetraspanins CD9, CD63, CD81, or CD82. Microvesicles (30–1000 nm in size) originate through evagination of the cytoplasmic membrane driven by a regulated mechanism, and are characterized by the presence of mitofilin, actin-4, and GP96. Apoptotic bodies (500–5000 nm in size) are generated during the late stage of apoptosis and are characterized by the exposition of phosphatidylserine at the outer surface (Díaz-Godínez et al. 2022). In addition to playing a critical role in cellular intercommunication, EVs have also been involved in several biological functions such as differentiation, proliferation, immune response, and metabolism regulation (Doyle and Wang 2019; Díaz-Godínez et al. 2022). Such a functional diversity has positioned EVs as a novelty strategy to develop acellular vaccines and treatments in different diseases, especially based on their immunogenic and immunoregulatory functions, respectively (Sabanovic et al. 2021; Santos and Almeida 2021).

In this review, we update the information on the use of EVs in the prognosis, treatment, and vaccination against COVID-19 generated so far in the pandemic. The collected data show that EVs are an important tool for the future development of strategies that lead to COVID-19 control.

## EVs as prognosis marker in COVID-19

More biomarkers are urgently needed to assess the risk and prognosis in COVID 19. Because EVs are present in all biological fluids, including blood, saliva, sputum, tears, and cerebrospinal fluid, they have been used as biomarkers for several human diseases. (Pulliero et al. 2019). Very early in the pandemic, SARS-CoV-2 infection was shown to be associated with the release, into the blood, of EVs carrying tissular factor that activates platelets and endothelial cells, thus contributing the EVs to the COVID-19-related thrombosis in patients (Mackman et al. 2020). The comparison between the proteomes of EVs from COVID-19 patients and EVs from healthy donors identified some EV-associated markers of SARS-CoV-2 infection, among which the coat complex subunit beta 2 (COPB2) results to be the best predictor to discern between mild and severe cases (Fujita et al. 2021). Moreover, SARS-CoV-2 RNA was also identified in the cargo of circulating EVs, suggesting that this virus may use the endocytosis route as Trojan horse to spread from one cell to another; nevertheless, viral RNA contamination during the EVs isolation process cannot be ruled out as reported elsewhere (Kongsomros et al. 2022). Therefore, circulating EVs may play a role in immunomodulation, inflammation, and coagulation during SARS-CoV-2 infection (Barberis et al. 2021).

On the other hand, platelet-derived EVs (PLT-EVs) are the most abundant circulating EVs in bloodstream (Berckmans et al. 2019). Activated platelets release PLT-EVs into the circulatory system, enhancing damage and inflammation in lung and other several organs (Manfredi et al. 2022). Moreover, PLT-EVs release has been shown to increase in several human diseases, including viral infections (Boilard et al. 2014; Rozmyslowicz et al. 2003). For instance, the combination of SARS-CoV-2 and human platelets induced platelet activation and increased PLT-EVs secretion (Maugeri et al. 2022), which has been related to poor prognosis in SARS-CoV-2-infected patients (Traby et al. 2022). A cohort of SARS-CoV-2-infected patients enrolled during both the first (April/May) and the second (October/November) pandemic waves showed high counts of PLT-EVs compared to SARS-CoV-2 negative controls (Cappellano et al. 2021). The same study showed through a ROC curve analysis that PLT-EVs are a good diagnostic marker of SARS-Cov-2 infection (Cappellano et al. 2021). Raineri et al. (2022) also observed higher counts of PLT-EVs in patients with SARS-CoV-22 infection compared to healthy controls; moreover, they also detected that during hospital stay, SARS-CoV-2-infected patients with poor prognoses had significantly increased PLT-EV counts, suggesting that the abundance of PLT-EVs could also be used as an indicator of SARS-CoV-2 infection severity (Raineri et al. 2022). Likewise, the counts of PLT-EVs increased in SARS-CoV-2-infected patients 30-day post-discharge, and the high abundance of PLT-EVs was associated with developing venous thromboembolism (Campello et al. 2022). However, another study found that both severe and non-severe SARS-CoV-2 patients presented a high abundance of PLT-EVs compared to healthy controls with no difference between infected groups (Zaid et al. 2020). Therefore, evidence indicates that PLT-EVs is a good diagnostic marker of SARS-CoV-2 infection, and probably, of poor prognosis; however, further studies are necessary to assess this latter proposal (all this information is summarized in Table 1).

**Table 1:** Summary of candidate EV prognosis markers of COVID-19 severity

EV origin	EV source	EV markers	Association with clinical outcomes	Reference
Platelet	Plasma	CD41^+^/Annexin V^+^CD41^+^	Increased in COVID-19 non-severe cases, but not in severe cases	Zaid et al. 2020
Platelet	Plasma	CD41a^+^/CD31^+^	Predictor of SARS-CoV-2 infection (75% sensitivity and 74% specificity)	Cappellano et al. 2021
Platelet	Plasma	HMGB1^+^	Independent predictor of the COVID-19 severity	Maugeri et al. 2022
Platelet	Blood	CD41a^+^/CD31^+^	Increase as biomarker of COVID-19 severity	Raineri et al. 2022
Platelet Leukocytes	Blood	P-Selectin^+^, E-Selectin^+^ and CD45^+^	P-Selectin^+^ EVs > 1,054/µL were associated with thrombosis, E-Selectin^+^ EVs ≤ 531/µL with worsening/death and 30-day P-Selectin^+^ and CD45^+^ EVs with persistent symptoms	Campello et al. 2022

## EVs as treatment for COVID-19

**Fig. 1:**
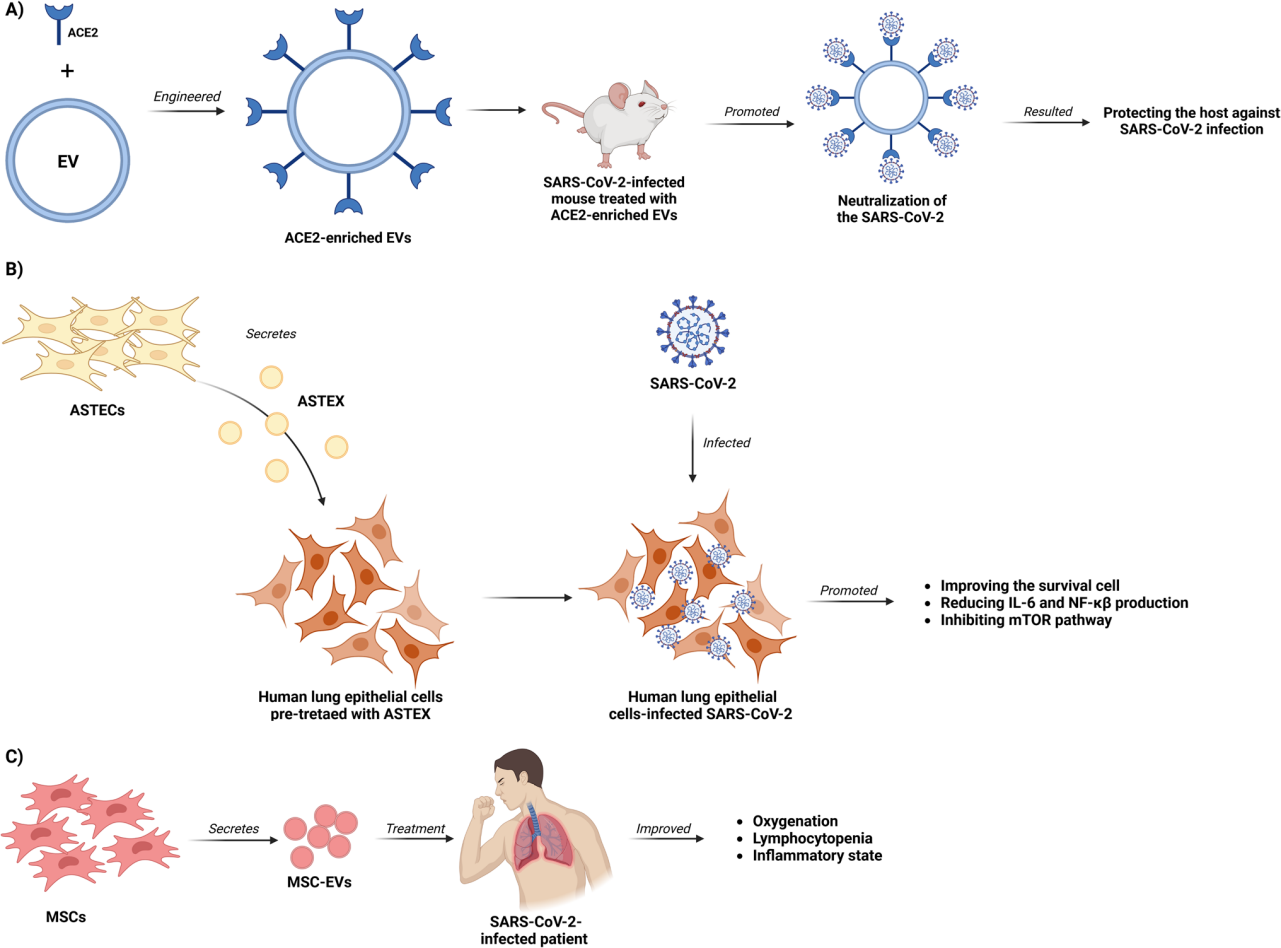
Summary of EV-based strategies proposed to control COVID-19 infection. **A** Use of EVs displaying the ACE2 receptor on their surface to neutralize SARS-CoV-2. **B** Use of ASTEX (activated specialized tissue effector EVs) to modulate the inflammatory response associated with the disease. **C** Use of mesenchymal stem cell-derived EVs (MSC-EVs) for the intranasal treatment of patients in order to attenuate the clinical signs of the disease

EVs are considered a novel mediator of intercellular communication due to their ability to transfer to recipient cells a large range of cargoes, including proteins, lipids, and nucleic acids, regulating thus the physiological and pathological functions throughout the body (Zhou et al. 2020). Therefore, EVs are considered the ideal carriers for delivering to cells active molecules and drugs for treatment of various diseases (Dang et al. 2020). Several studies have been done in this regard on SARS-CoV-2 infection (summarized in Fig. 1). For example, a recent study showed that ACE2-containing EVs bind to SARS-CoV-2 through the virus spike (S) protein (Zhang et al. 2021), promoting a decrease in SAR-CoV-2-S-pseudotyped virus infection in vitro (Cocozza et al. 2020). Hence, various studies have focused on engineering EVs enriched with the ACE2 protein as a plausible treatment against SARS-CoV-2 infection. Xie et al. (2021) designed SARS-CoV-2 blocking EVs by fusing the ACE2 sequence to the S-palmitoylation-dependent plasma membrane targeting sequence, resulting in EVs enriched with ACE2 on their surface. This modification in the membrane of EVs allowed the neutralization of the SARS-CoV-2 virus in human ACE2 transgenic mice, resulting in an efficient decrease of SARS-CoV-2 viral loads, and thus protecting the mice against SARS-CoV-2-induced lung inflammation. In another similar trial, Wu et al. (2022) reported that the intranasal pre-treatment of mice with engineered EVs stably expressing full-length human ACE2 (EVs-ACE2) blocked infection of nasal epithelium by S-pseudovirus. However, these findings must be taken with caution, as a report from the group of Tey et al. (2022) demonstrated that engineered EVs-ACE2 increased the infectivity of SARS-CoV-2 by enhancing viral entry into host cells in a much more efficient manner than viruses alone, serving as a “Trojan horse.” Therefore, more studies are required to elucidate the therapeutic role of ACE2-enriched EVs in SARS-CoV-2 infection.

On the other hand, Scott et al. (2022) proposed a molecular therapy against COVID-19 based on engineered EVs containing a fusion protein of CD63 with an anti-CoV-2 nanobody that bound SARS-CoV-2 spike at the receptor-binding domain (RBD) and exerted functional SARS-CoV-2 neutralization activity. Engineered neonatal fibroblasts used as factories for the production of therapeutic activated specialized tissue effector EVs (ASTEX) have also been assessed. Thus, the pre-treatment of human lung epithelial cells with ASTEX resulted in cytoprotective activity by improving cell survival through the reduction of IL-6 and NF-κβ production in 2-day post-infection with SARS-CoV-2 (Ibrahim et al. 2022). The same study found that ASTEX pre-treatment impaired SARS-CoV-2 infection by inhibiting the mTOR pathway (Ibrahim et al. 2022). Overall, the available evidence indicates that therapy with engineered EVs could be a plausible strategy for protection against SARS-CoV-2 infection. However, more clinical trials are needed to have better certainty about the efficacy of this type of molecular therapy in variable conditions of COVID-19 infection.

Another possibility of COVID-19 treatment based on EVs is the use of mesenchymal stem cell-derived extracellular vesicles (MSC-EVs), considered very promising nanotherapeutic agents. In this regard, a recent study demonstrated that MSC-EV treatment of SARS-CoV-2-infected human lung epithelial cells led to suppression of replication and release of SARS-CoV-2 viral particles from these cells (Chutipongtanate et al. 2022). Very interesting is the fact that the intravenous administration of MSC-EV in patients infected with SARS-CoV-2 resulted in a marked improvement of oxygenation within 3 days of treatment, alongside with reduction in neutrophil count and increase in lymphocyte count, as well as decrease in C-reactive protein (CRP), ferritin, and d-dimer levels (Sengupta et al. 2020). Likewise, inhaling MSC-EVs as a noninvasive strategy has emerged as a potential treatment for acute lung injury (ALI) induced by SARS-CoV-2 infection. Zhao et al. (2022) compared in mice the effects of MSC-EVs administration by inhalation and by tail vein injection for the treatment of ALI and found that inhalation of MSC-EVs reduced the expression of pro-inflammatory cytokines and the pathological scores, while simultaneously increased the expression of anti-inflammatory cytokines by polarizing macrophages towards M2 phenotype. In a pilot study with severe SARS-CoV-2 infection patients, it was found that the nebulization of human adipose-derived MSC-EVs improved the lymphocytopenia and a trend towards CRP, interleukin-6, and lactate dehydrogenase decrease, and different degrees of resolution of pulmonary lesions were observed (Zhu et al. 2022). Together, the evidence suggests that the use of MSC-EVs is a promising novel treatment for COVID-19.

## EVs and vaccination against COVID-19

The rapid spread of SARS-CoV-2 and the high mortality rate of COVID-19 during the first year of the pandemic highlighted the need to rapidly develop vaccines against this pathogen to protect the population (Halstead and Katzelnick 2020). After several months since the first cases were reported, and representing an unprecedent record, different types of vaccines with the ability to induce humoral and cellular responses against SARS-CoV-2 were put into circulation. Currently, there is a wide variety of vaccines that have been applied to people: messenger RNA vaccines (Pfizer-BioNTech, Moderna), viral vector vaccines (Oxford-AstraZeneca, CanSino Biologics, Johnson & Johnson), inactivated complete virus vaccines (Sinovac, Sinopharm), and vaccines made from viral subunits (Novavax) (Meo et al. 2021; Fathizadeh et al. 2021; Fiolet et al. 2022). All of them have shown their effectiveness by reducing the number of cases of severe disease; however, a vaccine that prevents infection in 100% of cases has yet not been developed.

**Fig. 2:**
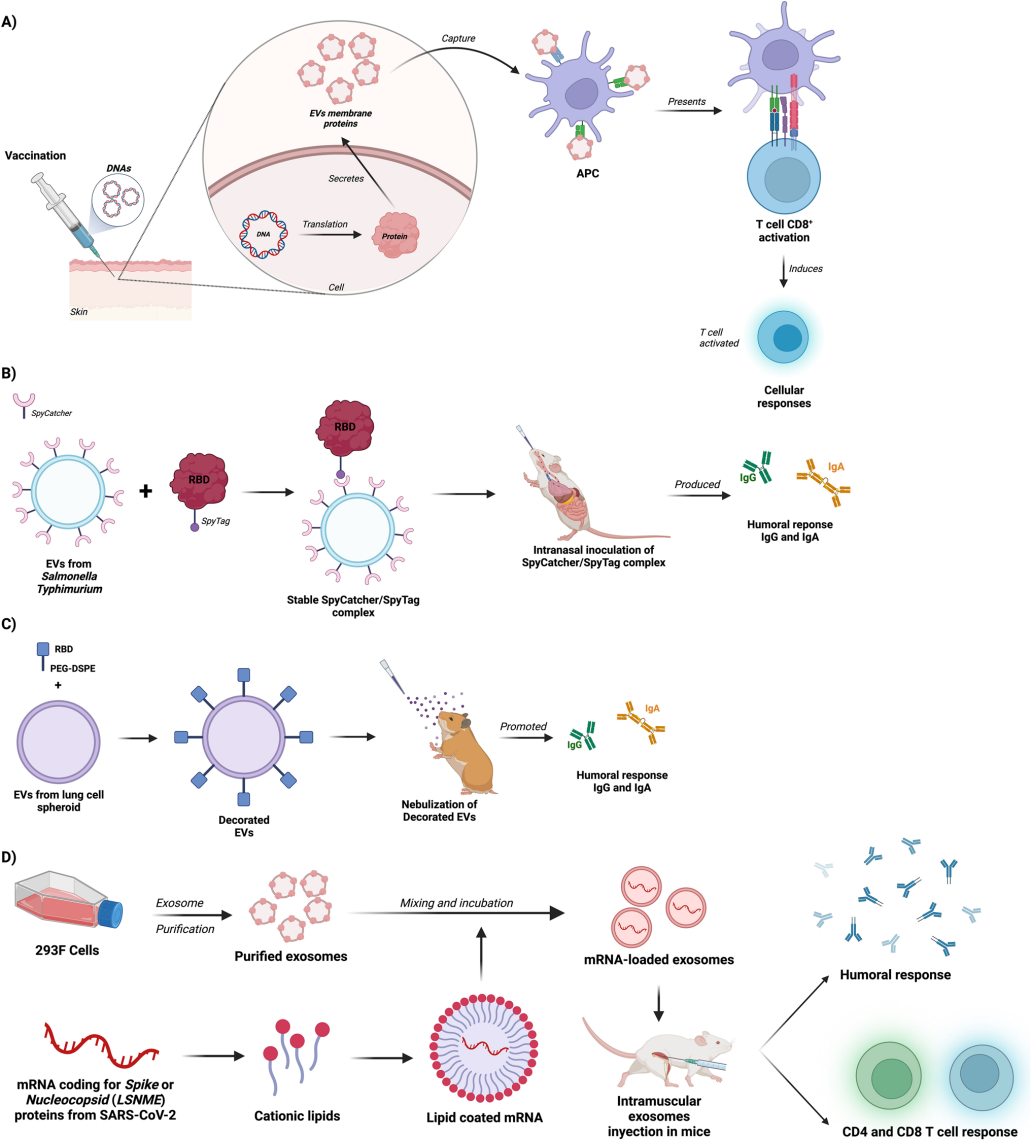
Summary of proposed EV-based strategies of vaccination against COVID-19. **A** Vaccination with DNA coding for SARS-CoV-2 structural proteins that are coupled to the EVs released by muscle cells. These EVs are captured by antigen presenting cells (APC) and the virus proteins presented to activate specific CD8 + T cells responses. **B** Vaccination with EVs displaying a SpyCatcher that captures the RBD of protein S (S-RBD) through a SpyTag. Inoculation with these EVs is intended to induce strong humoral responses that neutralize the virus. **C** Intranasal nebulization vaccination with EVs derived from lung spheroids showing the S-RBD coupled to DSPE (1,2-distearoyl-sn-glycero-3-phosphoethanolamine-poly(ethy lene-glycol)-N-hydroxysuccinimide) to induce neutralizing mucosal antibody responses. **D** Intramuscular vaccination with mRNA coding for Spike or nucleocapsid proteins loaded into exosomes. Inoculation induces humoral response and cellular immunity mediated by CD4 and CD8 lymphocytes

Different research groups have reported the use of EVs released from engineered pathogens as a simple and practical strategy of vaccination. In this regard, it has been demonstrated that EVs from engineered *Staphylococcus aureus* (Wang, et al. 2018), *Cryptococcus neoformans* (Rizzo et al. 2021), and *Streptococcus equi* (Lee et al. 2021) lead to a strong immune response reflected in decreased death rates of infected animals. The potential of EVs as vaccines is not limited to those produced naturally by some cell types, but they can also be engineered with biotechnological tools according to the intended purpose (Dooley et al. 2021). Here, we highlight 4 of the most recent strategies used to obtain EVs for vaccine purposes against COVID-19 (summarized in Fig. 2).

Ferrantelli et al. (2022) described a novel technique to provide cellular immunity based on EVs. They designed a DNA vector that drive the expression of the HIV-1 mutant protein Nef (Nef^mut^) which can be fused with other proteins and incorporated into EVs, which can then be released by different cell types (Muratori et al. 2009). The authors fused Nef^mut^ with structural SARS-CoV-2 proteins (S1, S2, M, and N) and, after transfection in HEK 239 T cells, obtained a high expression of the fusion proteins into the EVs released to the culture media (Ferrantelli et al. 2021). Intramuscular immunization of mice with this fusion vector triggered the production of SARS-CoV-2 specific CD8 + T cells with the Th1 profile (production of INF-γ and TNF-α), which were detected in the spleens, bronchoalveolar washings, and lungs of the immunized animals. In protection assays, the mice inoculated with the vector driving the expression of Nef^mut^-N protein showed greater resistance against SARS-CoV-2 infection compared to mice immunized with the control vector or the vector driving expression of Nef^mut^-S1 protein (Ferrantelli et al. 2022). The authors theorize that EVs released from muscle fibers after vector inoculation drive the expression of the fused proteins, which are taken up by antigen-presenting cells and presented to CD8 + T cells to trigger the specific response. This methodology aimed to induce a specific cellular immune response is of vital importance since the development of cellular immunity mediated by cytotoxic T lymphocytes has been associated with a good prognosis during COVID-19 (Li et al. 2020). Moreover, this strategy could provide a protective response even in patients that lack or have a poor humoral response (Kong et al. 2020).

A completely different strategy was used by Jiang et al. (2022) to trigger a systemic and mucosal humoral response. They obtained EVs from a detoxified mutant strain of *Salmonella typhimurium* which expressed the SpyCatcher peptide capable of binding to a SpyTag-containing protein. These EVs were purified from the bacterial culture and challenged with the recombinant Spike receptor binding domain (S-RBD) protein from SARS-CoV-2 fused to SpyTag. The interaction gave rise to S-RBD-decorated EVs (S-RBD-EV) due to the stable interaction between SpyCatcher present in the EV and the SpyTag of the RBD recombinant protein. According to the results obtained by this group, the intranasal immunization of mice with EV-S-RBD induced a humoral response represented by circulating and mucosal IgG antibodies with SARS-CoV-2 neutralizing activity. Contrary to that, the production of IgA and IgM antibodies in bronchoalveolar lavage was just above the limit of detection. In addition, S-RBD-EV inoculation reduced viral load and disease-associated histopathology in an experimental mouse model of SARS-CoV-2 infection.

Wang et al. (2022a) carried out a similar strategy to obtain EVs for vaccination purposes. This group conjugated the recombinant RBD protein from SARS-CoV-2 to 1,2-distearoyl-sn-glycero-3-phosphoethanolamine-poly(ethy lene-glycol)-N-hydroxysuccinimide, and bound this constructed molecule to exosomes derived from lung cell spheroids. The conjugated protein was successfully incorporated into the surface of lung exosomes allowing its use for immunization studies. The administration of these constructed EVs by nebulization in a golden hamster model led to the production of circulating IgG and mucosal IgG and IgA anti-SARS-CoV-2 RBD antibodies. In addition, the immunization with these EVs induced a cellular immunity mediated by CD4 + and CD8 + T lymphocytes that produce INF-γ, a Th1 response profile. The challenge of the immunized animals with SARS-CoV-2 demonstrated the effectiveness of this vaccine in reducing the viral load, as well as the inflammatory response in the lung. An additional feature of this vaccine is its ability to be freeze-dried and stored for up to 3 months without the need for a cold chain, which is the limitation for the transport of other currently used vaccines (Rusnack 2021).

Tsai et al. (2021) combined the use of mRNA and exosomes to create a vaccine with promising results. They obtained and purified exosomes from the 293F cell line and loaded them with mRNA encoding the Spike protein (S^W1^) from SARS-CoV-2. In addition, they also obtained exosomes loaded with mRNA encoding LSNME, a fusion of SARS-CoV-2 nucleocapsid, and fragments of Spike, membrane and enveloped proteins bound to the human Lamp 1 protein. To load the exosomes with mRNA, the authors treated the mRNA with cationic lipids and then incubated it with the exosomes. The intramuscular injection of the mRNA-loaded exosomes into C57BL/6 J mice induced the production of antibodies against the S and N proteins, which persisted through 3-month of the study, and the activation of reactive CD4^+^ and CD8^+^ T lymphocytes (Tsai et al. 2021). The cellular response against the S protein was associated with a Th1 profile characterized by high levels of INF-γ with low production of IL-4; however, the response against the N protein did not induce the production of any cytokine. Finally, the administration of the vaccine did not show adverse effects in the immunized animals (Tsai et al. 2021).

These data indicate the effectiveness of EVs-based vaccines to trigger immunity against SARS-CoV-2 that together with preclinical observations suggest that these vaccines could move on to the next phase of study.

## Concluding remarks and future directions

As an unprecedented event in the history of humanity, the arrival of the COVID 19 pandemic encouraged in a very short time the development of great advances in all aspects of the disease, but above all, in those related to its control. This minireview summarized the findings that have been obtained in these three years concerning the use of EVs for the control of SARS-CoV-2 infection. Although many unanswered questions and methodological challenges remain, this rapidly advancing field of EVs application will ultimately provide important insights into the relevance of EVs in the clinical setting of COVID-19. Rather than being useful for diagnosis, for which a wide variety of tests are available, studies to date on EVs produced during infection seem to indicate that their abundance in blood, detectable through the overexpression of some markers such as COPB2, could be used as a predictor of the severity of the infection. In this regard, the high levels of some exosomal miRNAs in the blood have been associated with a poor prognosis for some cancers, such as miR-19a for gastric cancer (Matsumura et al. 2015) and miR-1290 and miR-375 for castration resistant prostate cancer (Huang et al. 2015). In contrast, low levels of the exosomal miRNA miR-125b were associated with advanced melanoma (Alegre et al. 2014), suggesting that upward variations are not always the predictors. Studies of this type have not yet been carried out in patients with COVID 19, but they are necessary, as they could provide valuable information for the identification of severe cases, possible monitoring of the evolution of patients, and the use of miRNA-based therapeutics to reduce the severity of the illness. EVs arise as the appropriate delivery vehicle for these small non-coding RNAs, which could be envisioned as a promising biomarker in designing a practical iRNA-based treatment approach of clinical significance.

Perhaps the most interesting aspect of EVs is the possibility of using them as a carrier system for therapeutic targeting, drug delivery, and vaccination, through the intelligent design of EVs on demand and strategies of incorporation of useful molecules to their membranes or interior. In this sense, the possibilities are unlimited and are yielding very interesting results, including for SARS-CoV-2 infection. In particular, the use of SARS-CoV-2 blocking EVs displaying the ACE2 receptor has proved to be efficient in neutralizing the virus in vitro and inhibiting pulmonary inflammation in a murine model of infection, and EVs from neonatal fibroblasts (ASTEX) were both cytoprotective and inhibited the viral infection. A rather interesting option that has not yet been explored would be the use of EVs encapsulating antiviral siRNA (small interfering RNA) to inhibit SARS-CoV-2. This strategy has been used successfully to inhibit the vertical transmission of Zika virus in a mouse model, helping to alleviate virus-induced microcephaly by delivering the siRNA to the fetus brain (Zhang et al. 2022A, B). Finally, it is noteworthy to mention that EVs may represent the best cost-effective option for vaccination as they are increasingly easy to obtain, exhibit a low basal immunogenic profile, can be engineered to display specific CD8( +) T cell and B cell viral antigens, and can induce a strong immune response. Therefore, engineered EVs represent an efficient, flexible, and safe strategy for a virus-free vaccine design. In fact, this is the goal of some biotech companies such as Capricor Terapeuthics, Ciloa Company, and Codiak BioSciences, who are developing EV-based vaccines for COVID-19, by displaying the SARS-CoV-2 structural proteins on the EVs surface or by delivering their mRNAs through them (reviewed in Sabanovic et al 2021). All this augurs a promising future for the use of engineered EVs for various purposes, for the definitive control not only of COVID-19 but also of any other infectious disease.
